# *STK39 *polymorphisms and blood pressure: an association study in British Caucasians and assessment of *cis*-acting influences on gene expression

**DOI:** 10.1186/1471-2350-10-135

**Published:** 2009-12-14

**Authors:** Michael S Cunnington, Chris Kay, Peter J Avery, Bongani M Mayosi, Mauro Santibanez Koref, Bernard Keavney

**Affiliations:** 1 Institute of Human Genetics, Newcastle University, Central Parkway, Newcastle upon Tyne, NE1 3BZ, UK; 2 Department of Medicine, University of Cape Town, Cape Town, South Africa

## Abstract

**Background:**

Blood pressure (BP) has significant heritability, but the genes responsible remain largely unknown. Single nucleotide polymorphisms (SNPs) at the *STK39 *locus were recently associated with hypertension by genome-wide association in an Amish population; *in vitro *data from transient transfection experiments using reporter constructs suggested that altered *STK39 *expression might mediate the effect. However, other large studies have not implicated *STK39 *in hypertension. We determined whether reported SNPs influenced *STK39 *expression *in vivo*, or were associated with BP in a large British Caucasian cohort.

**Methods:**

1372 members of 247 Caucasian families ascertained through a hypertensive proband were genotyped for reported risk variants in *STK39 *(rs6749447, rs3754777, rs35929607) using Sequenom technology. MERLIN software was used for family-based association testing. *Cis*-acting influences on expression were assessed *in vivo *using allelic expression ratios in cDNA from peripheral blood cells in 35 South African individuals heterozygous for a transcribed SNP in *STK39 *(rs1061471) and quantified by mass spectrometry (Sequenom).

**Results:**

No significant association was seen between the SNPs tested and systolic or diastolic BP in clinic or ambulatory measurements (all p > 0.05). The tested SNPs were all associated with allelic expression differences in peripheral blood cells (p < 0.05), with the most significant association for the intronic SNP rs6749447 (P = 9.9 × 10^-4^). In individuals who were heterozygous for this SNP, on average the G allele showed 13% overexpression compared to the T allele.

**Conclusions:**

*STK39 *expression is modified by polymorphisms acting in *cis *and the typed SNPs are associated with allelic expression of this gene, but there is no evidence for an association with BP in a British Caucasian cohort.

## Background

Hypertension is a major risk factor for vascular and renal disease and thereby contributes to substantial worldwide morbidity and mortality [1]. Although blood pressure (BP) has been shown to have significant heritability (30-60%) [2], the genes conferring susceptibility to hypertension remain largely unknown. A recent genome-wide association study (GWAS) analysed 79,447 single-nucleotide polymorphisms (SNPs) in the American Old Order Amish, a closed population descended from a small number of common founders who emigrated from Switzerland in the early 1700s who have a relatively homogeneous lifestyle [3]. Their initial genome-wide screen of 542 subjects from the Amish Family Diabetes Study [4], in which families were ascertained through a proband with type 2 diabetes, identified a cluster of SNPs in the *STK39 *(serine threonine kinase 39) locus on chromosome 2q24.3 that were associated with BP (P = 8.9 × 10^-6 ^to 9.1 × 10^-5^) [3]. Although the P-value did not reach the conventionally accepted threshold for genome-wide significance [5], the inclusion of additional data from an Amish and four non-Amish Caucasian cohorts led to a significant result (P = 1.6 × 10^-7^) in the combined dataset of 7,125 individuals. The replication cohorts included a further population of 2,842 individuals from a case-control study of diabetes and three smaller groups not selected for diabetes.

The *STK39 *gene encodes the SPAK (Ste20-related proline-alanine-rich kinase) protein which interacts with ion cotransporters involved in salt transport and osmotic cell volume regulation, including the thiazide-sensitive and loop diuretic-sensitive cotransporters involved in renal salt excretion [6,7]. The *STK39 *gene contains 18 exons spanning approximately 300 kb on chromosome 2q24.3; SNPs associated with BP by GWAS were located within introns 1-8, with no coding or splice variants identified by sequencing [3]. Using transfection experiments with luciferase reporter constructs Wang and colleagues demonstrated that alleles of one SNP (rs35929607), located in a conserved region of intron 2, altered expression of the reporter constructs *in vitro*. This SNP was in linkage disequilibrium (LD) with the SNPs identified by GWAS, suggesting a possible functional mechanism for the observed association.

Despite these results, other large GWAS and meta-analyses involving up to 71,225 Caucasian individuals have failed to identify association between *STK39 *SNPs and BP that satisfies genome-wide significance thresholds [8-10]. However, such data do not exclude an effect of this locus on BP, since the statistical threshold for replicating an association differs from the threshold for genome-wide 'discovery'. Differences in the actual SNPs genotyped, patterns of LD, and phenotypic variation between populations might contribute to the lack of genome-wide significance in these studies, compared to the GWAS by Wang and colleagues. One recent GWAS looked specifically for evidence of correlation between *STK39 *SNPs and BP in 1017 African American subjects and provided some supportive evidence for an association [11]. SNPs in the *STK39 *region did not reach genome-wide significance, but the number of significantly associated SNPs at a nominal p threshold of p < 0.05 was somewhat higher than expected by chance alone (9/136 for systolic BP and 33/136 for diastolic BP, compared to 7/136 expected by chance).

We aimed to test whether the *STK39 *SNPs identified in the report by Wang and colleagues [3] were associated with BP in a large family-based Caucasian cohort ascertained through probands with essential hypertension [12,13]. This cohort has a higher average BP (134 v 128 mmHg systolic) and lower proportion of diabetics (2.5 v 22.2%) compared to the Amish population used for the initial genome-wide study by Wang and colleagues, but has 24-hour ambulatory BP recordings which provide a more reproducible and precise measure of true BP, with better prediction of cardiovascular events, compared to clinic BP measures [14,15]. Selecting the same SNPs typed by Wang and colleagues and using a cohort enriched for hypertension with ambulatory BP data should increase the power to detect effects associated with these variants in our population.

We also aimed to confirm whether the SNPs have *cis*-acting effects on *STK39 *expression *in vivo *using allelic expression imbalance (AEI). This technique measures the relative amount of transcript arising from each allele in individuals heterozygous for a transcribed polymorphism. Unequal amounts of transcript from each allele indicate the presence of *cis*-acting effects on gene expression [16]. The allelic expression ratio can be used to quantify the degree of AEI and test whether particular SNPs are associated with *cis*-acting effects, even in the absence of LD between the transcribed and *cis*-acting SNPs [17]. The degree to which data from transfection studies in cell lines represents the *in vivo *situation in complex human tissues is questionable [18]. AEI assesses alleles expressed in their native environment, and because comparison of alleles is made within samples, *trans*-acting influences and experimental insults are the same for each allele which maximises the sensitivity for detecting *cis*-acting effects [16].

## Methods

### Association of SNPs with blood pressure

#### Family ascertainment

1372 members of 247 British Caucasian families ascertained through hypertensive probands and phenotyped for a quantitative genetic study of cardiovascular risk factors from 1993-2001 were included. A detailed description of this series and the ascertainment strategy has been published previously [12]. Briefly, families were selected through a proband with essential hypertension whose systolic and diastolic BP were in the top 5% of the population distribution (defined as daytime ambulatory BP >140/90 mmHg; three clinic BP measurements >160/95 mmHg; or treatment with at least two antihypertensive medications). Secondary hypertension was excluded using the screening protocol applied in the hypertension clinic. Families were required to consist of at least three siblings (including the proband) clinically assessable for BP if DNA from a parent of the sibship was available, or at least four siblings if no parental DNA was available. Qualifying sibships could be in the generation of the proband, or the offspring. There was no requirement for additional members of the family to be hypertensive, but where additional members of the sibship were found to have hypertension (using the same criteria), families were extended and the spouses and offspring of hypertensive members also collected. The majority (64%) of the individuals in the family collection therefore have BP within the conventionally accepted "normal range", and the family collection includes some extended families, though most are nuclear families. A total of 1425 subjects from 248 families were recruited; all 1372 family members in whom DNA was available were genotyped in the present study. The median family size was 5 people, 60% of families comprising between 4 and 6 genotyped and phenotyped members. 71% of families were 2-generation and 29% were 3-generation. 84% of families had an assessable sibship in the generation of the proband, while 16% of families consisted of a proband and their nuclear family (spouse and children over 18 years) only. The study complies with the principles of the Declaration of Helsinki; informed consent was obtained from all participants and the study was approved by the Central Oxford Research Ethics Committee and Newcastle and North Tyneside Local Research Ethics Committee.

#### BP measurement

Blood pressure was measured using ambulatory monitoring for a period of 24 hours in all subjects willing to undergo monitoring, using the A&D TM2421 monitor. Three readings were taken with the patient in a relaxed seated position at the start of the monitoring period. Simultaneous auscultation was carried out by a trained observer, to confirm satisfactory (within 5 mmHg) agreement between the monitor and auscultatory values; if this criterion was not met, the cuff was repositioned until satisfactory agreement was obtained. The three readings which had satisfactory agreement between the monitor and the observer in the final cuff position are referred to as "clinic readings". The monitor was programmed to record blood pressure every half-hour during the daytime and every hour during the night, and a recording was considered of satisfactory technical quality if at least 20 daytime ambulatory data points were available for analysis. Patients also recorded the time they went to bed and rose in the morning to enable individualised calculation of the "daytime" and "night-time" periods. Mean values for systolic and diastolic blood pressures for the clinic, daytime and night-time periods were analysed for association with genotypes.

A full clinical history was taken, which included the subject's medical history and lifestyle factors including consumption of alcohol and tobacco, and habitual physical exercise. Anthropometric data including height, weight and waist and hip circumferences were measured. DNA was extracted from blood samples using standard methods.

#### Genotyping

Four SNPs reported to be associated with hypertension by Wang and colleagues [3] were selected for genotyping; three at the *STK39 *locus on chromosome 2q24.3 (rs6749447, rs3754777 and rs35929607) and one on chromosome 9p21.3 (rs4977950) which gave the most significant signal for genome-wide association in the same study [3].

Multiplex SNP genotyping was performed using PCR followed by primer extension and MALDI-TOF mass spectrometry using iPLEX Gold technology from Sequenom (Sequenom Inc, San Diego, USA). SNP assays were designed using Sequenom's RealSNP http://www.RealSNP.com and MassARRAY Assay Design v3.0 Software. Primer sequences and full experimental methods are available in Additional File 1. Spectra were analysed using MassARRAY Typer v3.4 Software (Sequenom). Spectra and plots were manually reviewed and auto-calls were adjusted if required. Positive and negative controls were included. Mendelian inheritance and correspondence of genotype frequencies to Hardy-Weinberg proportions were tested using PEDSTATS [19].

### Statistical analysis

The analysis was performed using log transformed BP values to approximately normalise the distribution [12]. Adjustments were then made for significant covariates determined by linear regression, considering age, sex, cardiovascular medications, smoking status (current/former/never), alcohol consumption (units per week) and exercise habit (frequency per week). For those participants taking antihypertensive medication, the effects of the main drug classes (diuretics and β-blockers) were estimated from the data by regression, and the appropriate adjustment made to the on-treatment BP values, as previously described [12]. Association between genotypes and adjusted phenotypes was assessed using MERLIN v1.1.2 [20,21]. In order to estimate the upper bound of the genetic effect that is plausibly associated with each phenotype/SNP combination, linear regression models were fitted to model additive genetic effects using Minitab v15. Narrow-sense heritability estimates (and 95% confidence intervals thereof) were calculated by standard population genetic formulae. The narrow-sense heritability (h^2^) of each phenotype and proportion of phenotypic variation accounted for by covariates (r^2^) is shown in Table 1.

**Table 1:** Clinical and BP characteristics of the Caucasian participants

Characteristic	*N*	Median (LQ, UQ) in mmHg	Proportion of phenotypic variability (r^2^) explained by covariates for log-transformed variable	Heritability (h^2^) for adjusted variable
Hypertension	490	-	-	-
Current smoker	298	-	-	-
Diabetes	35	-	-	-
Structural heart disease	55	-	-	-
Cardiovascular medication	456	-	-	-
Clinic Systolic BP	1138	134 (121.3, 152.3)	27.2	11.2
Clinic Diastolic BP	1130	82 (73.7, 92)	19.7	12.2
Day Systolic BP	1134	132.5 (122.1, 146)	20.4	13.6
Day Diastolic BP	1133	79.7 (72.5, 90)	17.9	10.7
Night Systolic BP	903	113 (103.8, 126)	11.4	24.3
Night Diastolic BP	902	66 (60.2, 73.6)	13.6	34.6

N = 1372 Caucasian participants. BP, blood pressure; LQ, lower quartile; UQ, upper quartile.

### Association of SNPs with *STK39 *allelic expression

#### Identification of transcribed markers for allelic expression analysis

Allelic expression measures the relative amount of transcript arising from each allele in individuals heterozygous for a transcribed polymorphism. NCBI dbSNP (http://www.ncbi.nlm.nih.gov, 6^th ^January 2009) was used to identify SNPs in transcribed regions of *STK39 *with minor allele frequencies greater than 5%. Nine transcribed variants were identified (rs1061471, rs3769429, rs7425806, rs3769428, rs56330212, rs56697518, rs56031549, rs1802105, rs56048258). Only two of these had previously been validated according to dbSNP (minor alleles observed in at least two chromosomes): rs1061471 in exon 18 (3' untranslated region) and rs56031549 in exon 11 (missense A/G SNP). Three of the reported variants (rs7425806, rs3769429 and rs3769428) were non-polymorphic in all HapMap populations. SNP rs1061471 was non-polymorphic in the Caucasian HapMap CEU population, but had a minor allele frequency of 11% and informative heterozygote frequency of 24% in the African HapMap YRI population. In order to find transcribed markers suitable for assessing allelic expression, we analysed rs1061471 and the five markers for which HapMap population frequencies were not available (rs1802105, rs56031549, rs56048258, rs56330212, rs56697518) as described below.

#### Samples

For expression analysis we chose a cohort collected from an African population comprising 309 anonymous South African blood donors (self-reported ethnicity for these individuals was: Cape Mixed Ancestry 200; black African 67; Indian 30; white 10; other 2). The study complies with the principles of the Declaration of Helsinki; written informed consent was obtained from all participants and the study was approved by the University of Cape Town Faculty of Health Sciences Research Ethics Committee. Compared to a Caucasian population such a cohort is expected to exhibit greater genetic diversity, i.e. an increase of the probability of obtaining informative samples (heterozygous for at least one transcribed marker) and a reduction of the length of blocks with high LD. The latter will improve the ability to separate the effects of different SNPs on expression. Peripheral blood for DNA and RNA analysis DNA was extracted using an in-house phenol/chloroform method from 4 ml of peripheral blood in EDTA. Genotyping was performed on the Sequenom platform (as described above) for rs1061471 and the other five markers for which HapMap population frequencies were unknown (rs1802105, rs56031549, rs56048258, rs56330212, rs56697518). 35 individuals heterozygous for the transcribed variant rs1061471 and therefore suitable for allelic expression analysis were identified. The other five SNPs tested had minor allele frequencies <1%, giving an insufficient number of heterozygotes to be used for AEI assessment (Additional File 1 Table S2).

For the 35 individuals heterozygous for rs1061471, RNA was extracted from 2.5 ml of peripheral blood collected using the PAXgene system (Qiagen) without DNase treatment and eluted into a volume of 80 μl. The RNA solution was then DNase treated using RQ1 RNase-Free DNase (Promega). Mean total RNA concentration quantified by photospectrometry was 166 (± 65) ng/μl in the eluate. Approximately 2 μg of total RNA was reverse transcribed using the SuperScript VILO cDNA Synthesis Kit (Invitrogen) and eluted in 20 μl.

#### Measurement of *STK39 *allelic expression ratios

Quantification of the allelic expression ratio was performed by uniplex PCR from 1 ul of cDNA followed by primer extension and MALDI-TOF mass spectrometry using iPLEX Gold technology from Sequenom (Sequenom Inc, San Diego, USA). Primer sequences and full experimental methods for this are available in Additional File 1. Allelic ratios were estimated as the ratios of the area under G and A peaks, and were performed in four replicates from the same cDNA preparation. Results from amplification of genomic DNA (where alleles are present in a 1:1 ratio) from 19 individuals (performed in 4 replicates) were used as the equimolar reference to normalise the cDNA values.

#### Statistical analysis

Analyses were performed as described by Teare and colleagues [17] using the logarithm of the normalised allelic expression ratios.

## Results

### Association with blood pressure

1372 individuals were included; 649 (47%) were male, 490 (36%) were hypertensive, and mean age was 49 (standard deviation 15.5) years. The clinical characteristics and BP parameters of participants are shown in Table 1. Median alcohol consumption was 3 (interquartile range 0-12) units per week. 43% reported no regular exercise per week, with 21%, 22% and 14% exercising once, twice, and three or more times per week respectively.

Genotyping was complete for more than 99% of individuals at all loci. Allele frequencies for typed SNPs are shown in Table 2; these were similar to the HapMap CEU population and did not deviate significantly from Hardy-Weinberg proportions.

**Table 2:** Allele frequencies of the SNPs typed in study participants

SNP	Chromosome location	Alleles*	Number genotyped	HW P-value	Frequency of putative risk allele		
					
					Caucasian cohort	HapMap CEU	SA cohort
**rs3754777**	2q24 (*STK39 *intron)	G/**A**	1367	0.63	0.17	0.13	0.16
**rs35929607**	2q24 (*STK39 *intron)	A/**G**	1361	0.79	0.19	NA	0.34
**rs6749447**	2q24 (*STK39 *intron)	T/**G**	1369	0.32	0.29	0.28	0.52
**rs4977950**	9p21 (not within gene)	G/**C**	1365	0.46	0.15	0.18	NA

* Major allele given first, putative risk allele for hypertension identified by Wang et al shown in bold; MAF, minor allele frequency; CEU, HapMap CEU Caucasian cohort; NA, not available; SA, South African.

There was no significant association between genotype and covariate-adjusted, log transformed BP parameters at the loci studied, using a global P-value of p = 0.05. Estimates of effect size with 95% confidence intervals are shown in Table 3; the upper bound of the contribution of typed SNPs to the total population variance was low for all of the phenotypes tested (ranges: rs3754777 0.4-1.1%; rs35929607 0.5-1.7%; rs6749447 0.4-1.4%; rs4977950 0.4-0.6%).

**Table 3:** Effect sizes of tested SNPs

BP phenotype*	*N*	rs3754777	rs35929607	rs6749447	rs4977950
Clinic Systolic	1138	0.2 (-1.8, 2.3)	1 (-1, 2.9)	0.5 (-1.2, 2.2)	0 (-2.1, 2.1)
Clinic Diastolic	1130	-0.3 (-1.6, 1.1)	0.4 (-0.9, 1.7)	0.1 (-1, 1.2)	0.3 (-1.1,1.7)
Day Systolic	1134	-0.1 (-1.9, 1.7)	-0.3 (-1.5, 2)	0.1 (-1.4, 1.6)	0.2 (-1.7, 2)
Day Diastolic	1133	-0.1 (-1.4, 1.1)	0.2 (-1, 1.4)	0.1 (-0.9, 1.2)	0.4 (-0.9, 1.7)
Night Systolic	903	0.4 (-1.4, 2.1)	0.9 (-0.8, 2.6)	0.7 (-0.7, 2.2)	-0.2 (-2.1, 1.7)
Night Diastolic	902	0.7 (-0.5, 1.9)	1.1 (0, 2.3)	0.8 (-0.2, 1.8)	-0.1 (-1.3, 1.2)

* All phenotypes log-transformed to approximately normalise distributions and adjusted for covariates before analysis; Effect sizes are the mmHg difference attributable to 1 copy of the risk (minor) allele, with 95% confidence interval given in brackets. P-value > 0.05 for all effects.

### Association with allelic expression

**Figure 1:**
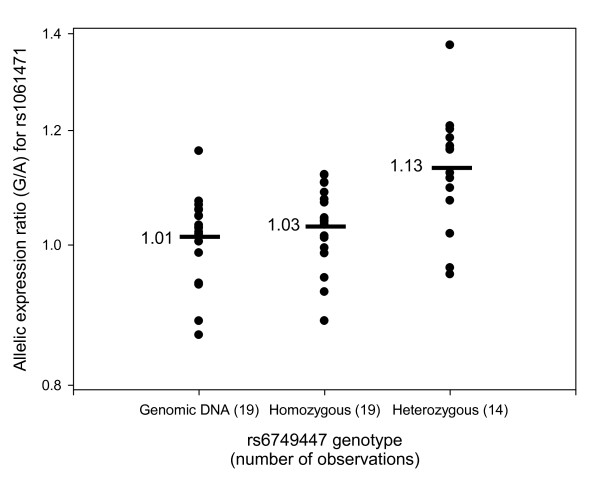
**Effect of genotype at rs6749447 on allelic expression ratio of the transcribed SNP rs1061471**. Circles represent allelic expression ratio for each individual, with horizontal bars representing the mean values for each group (shown alongside). The first column shows genomic DNA where the alleles are present in a 1:1 ratio, giving a mean ratio of approximately 1. The second column shows individuals who are homozygous for either allele of rs6749447. In this group, the *cis*-acting influence on expression from each allele is the same, giving a mean allelic expression ratio of approximately 1 at the transcribed marker rs1061471 (P > 0.05 for the comparison with genomic DNA). The third column shows individuals who are heterozygous for rs6749447. In this group each of the two transcribed alleles is expressed at a different level, causing increased imbalance in allelic expression (P < 0.005 for the comparisons with genomic DNA and individuals homozygous for rs6749447).

Two of the 35 samples failed quality control criteria for allelic expression measurements at the transcribed SNP rs1061471 because of high standard error between replicates and were excluded from the analysis; all replicates for the remaining 33 samples were included. The experimental variability was low and the average standard error of the log-transformed ratio for the four technical replicates was the same in gDNA (0.0146) and cDNA (0.0149). The standard deviation of the sample means (represented in Figure 1) was 0.030 in genomic DNA and 0.039 in cDNA.

**Figure 2:**
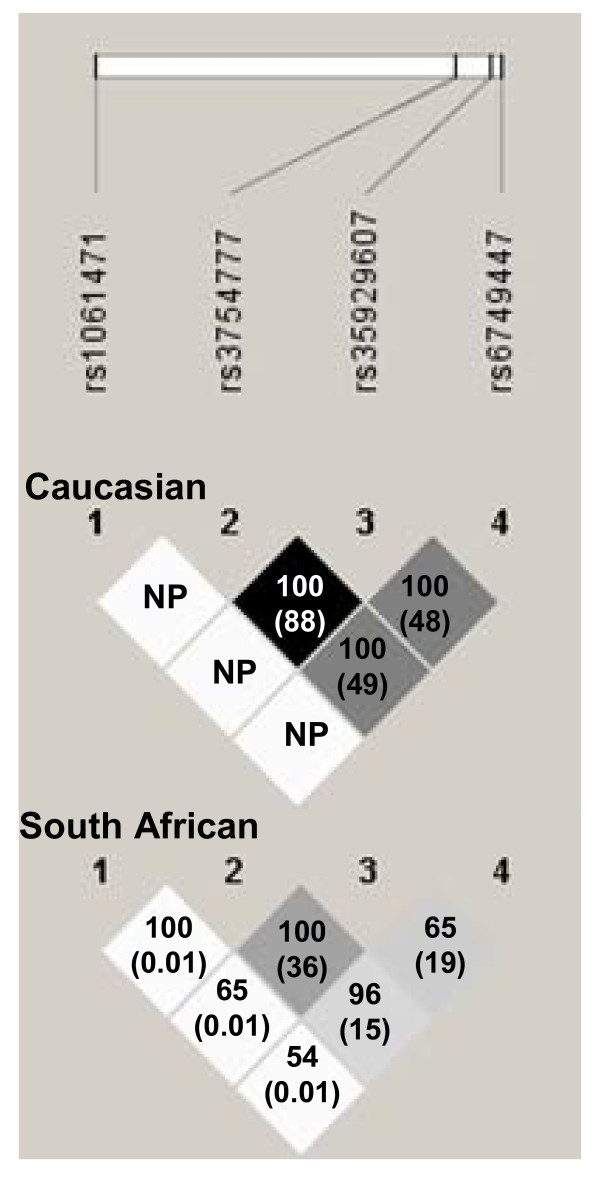
**Linkage disequilibrium between typed SNPs at the chromosome 2q24 *STK39 *locus in Caucasian and South African participants**. Shading represents r^2 ^values (r^2 ^= 0 white, 0 < r^2 ^< 1 shades of grey, r^2 ^= 1 black). Numbers show D' values (and r^2 ^values in brackets) between SNPs. NP, non-polymorphic in Caucasian population. Figure adapted from Haploview.

The tested SNPs were all significantly associated with allelic expression differences in peripheral blood, as shown in Table 4. The most significant association with expression was for rs6749447 (P = 9.9 × 10^-4^). This SNP is located in intron 1 of STK39 and in our SA sample had minor allele frequency 0.48 and D' 0.54 with the transcribed SNP rs1061471 located in the 3' untranslated region of the gene. Of the 33 included individuals who were heterozygous for the transcribed SNP rs1061471, 14 were heterozygous for rs6749447. If a SNP affects expression in *cis *then individuals heterozygous for that SNP should show a greater allelic imbalance at the transcribed SNP than homozygous individuals. Figure 1 shows the allelic expression ratios grouped by whether individuals are heterozygous or homozygous for rs6749447, compared to the ratio seen in genomic DNA (where alleles are present in a 1:1 ratio). There was no difference in allelic expression ratios between genomic DNA and individuals homozygous at rs6749447 (P = 0.07 using the Mann-Whitney U test). However, individuals heterozygous for rs6749447 have on average a higher allelic expression ratio than the homozygous individuals (P = 0.005 using the Mann-Whitney U test), which is consistent with a *cis*-acting effect and also suggests that the overexpressing allele is preferentially in phase with the G allele at the transcribed locus. In individuals who were heterozygous for rs6749447 on average the G allele of rs6749447 showed 13% overexpression compared to the T allele. Once the effect of this SNP was adjusted for, the association for the other SNPs no longer remained significant (as shown in Table 4). Differences in allele frequencies and patterns of linkage disequilibrium between the Caucasian and South African populations are summarised in Table 2 and Figure 2.

**Table 4:** Association of tested SNPs with allelic expression differences

SNP	P-value	P-value adjusted for rs6749447
rs35929607	0.02	0.39
rs3754777	0.006	0.71
rs6749447	0.001	-

## Discussion

The *STK39 *SNPs reported to be associated with BP and hypertension by Wang and colleagues [3] did not show a significant association with BP in our British Caucasian families. Other published GWAS and two large recent meta-analyses did not identify associations achieving genome-wide significance levels for *STK39 *polymorphisms and BP, despite powerful analyses of combined cohorts involving 34,433 and 71,225 Caucasian individuals which successfully identified multiple other susceptibility loci [9,10]. Although failure to achieve genome-wide significance levels does not exclude an effect of this locus on BP, any effect of sequence variation at *STK39 *on BP is too small to be detected in our data and is perhaps something particular to the Amish population. We also found no association with BP for the chromosome 9p21.3 SNP rs4977950 which was the top signal in the GWAS by Wang and colleagues (P = 9.1 × 10^-8^), and the only SNP to achieve significance at the genome-wide threshold in their study.

Inadequate power is an unlikely explanation for the lack of association in this study as the maximum plausible genetic effect for the typed SNPs on BP phenotypes, which we calculated based on the observed mean effect and 95% confidence intervals, was low in all cases and our data exclude an effect as large as the 3 mmHg increase in systolic BP per copy of the risk allele reported by Wang and colleagues in the Amish population. Families in the present study were selected through a hypertensive proband and had a higher proportion of hypertensive individuals and wider distribution of BP values; this should increase the power to detect an association with BP phenotypes compared to the cohorts in the study by Wang and colleagues, which were not selected for BP. The use of 24-hour ambulatory BP recordings in our study provides a more reproducible assessment of "usual" BP and reduces misclassification due to "white-coat" or masked hypertension that may occur with isolated clinic measurements [14,15]; this should also reduce the noise and increase the power of our study. The heritability of night-time BP measures was significantly higher than clinic BP measures, which should increase the power for detecting genetic effects. It is a frequently observed phenomenon in genetic epidemiology that studies first reporting a novel association often show a more extreme odds ratio than subsequent replication studies [22]; if the effect size estimated by Wang et al is an upwardly biased estimate this might explain the negative association in our study and the other reported GWAS. The effect due to *STK39 *alleles observed by Wang et al was strongest in the Amish population and substantially weaker in the other Caucasian cohorts tested in their study. Our data suggest that the effect of the typed *STK39 *SNPs on BP is at most modest in a UK Caucasian population. If the reported association was caused by LD between the typed SNPs and another functional variant, then weaker LD in other populations, compared to the Amish population, could account for the results; this is particularly pertinent since closed founder populations are expected to exhibit extensive LD. Heterogeneity between the populations, including differences in population history, recruitment strategy, and phenotypes (such as the proportion of diabetics and hypertensives) might also contribute to the differences in genetic associations we have observed. Our analysis was performed using an additive model because this was the model used to report the most significant overall association by Wang and colleagues.

We have demonstrated that SNPs in the *STK39 *gene correlate with *in vivo *allelic expression of this gene in peripheral blood cells. This corroborates and extends the findings from reporter gene constructs in the study by Wang and colleagues. Using a luciferase assay in HELA and HEK293 cell lines they examined the effect on transcription of two SNPs (rs12692877 and rs35929607) that are in conserved elements and which were in complete LD with two of the SNPs associated with BP in their GWAS in the Amish population (rs6749447 and rs3754777 respectively). They demonstrated that the G allele of rs35929607, which was the allele reported to be associated with increased BP, was in isolation associated with a greater than two-fold increase in transcriptional activity compared to the A allele of this SNP, or either allele of rs12692877. Such *in vitro *studies have limitations since results depend on the constructs that are used and expression is considered outside of the normal chromatin and cellular context, which may not reflect true expression in complex tissues *in vivo *[16,18]. Allelic expression analysis allows *in vivo *assessment of RNA transcripts in their native environment and regulatory context, and by controlling for *trans*-acting influences has high sensitivity to detect *cis*-acting effects. We found a stronger association between genotype and allelic expression for rs6749447 than for rs35929607, with the G allele of rs6749447 associated with a 13% increase in expression relative to the T allele. However, if the effect of rs6749447 is not accounted for, then our allelic expression analysis found that the G allele of rs35929607 was significantly associated with increased expression. This is consistent with the *in vitro *data from Wang and colleagues, although the magnitude of the relative increase in our study was lower at 7%. The difference in the magnitude of effect we observed compared to the *in vitro *transfection studies is not surprising in view of the fact that *in vivo *expression is likely to result from the interaction of multiple elements that modulate expression, rather than just a single SNP.

AEI can only be assessed in individuals who are heterozygous for a transcribed polymorphism in the gene of interest - there were no suitable transcribed SNPs in *STK39 *in Caucasian populations and in the African cohort the number of suitable heterozygotes was relatively small because of a low minor allele frequency (7%) at the transcribed SNP rs1061471. However, measurement of allelic expression ratios within samples is very sensitive for detection of *cis*-acting influences since *trans*-acting and experimental factors are identical for each allele (because comparisons are made within samples); this allows significant *cis*-acting effects to be detected in relatively few samples, as demonstrated in this study. Sensitivity of the technique for the detection of significant effects in equivalent sample sizes has been previously demonstrated [23,24]. Populations of African-descent show on average smaller regions of high LD and greater heterozygosity compared to Caucasian populations [25,26], and therefore using a South African cohort increases the sensitivity to separate the effects of different SNPs on expression. This trans-ethnic approach using populations of African descent has been shown to improve the fine mapping of functional polymorphisms associated with quantitative traits when strong LD between markers limits resolution of the functional locus in Caucasian populations [27]. In the case of *STK39*, using a population of different ethnicity allowed allelic expression to be measured in the absence of transcribed polymorphisms in the Caucasian population. Correlation between *STK39 *SNPs and expression of this gene does not necessarily mean that these SNPs will influence BP and the finding of an association with *STK39 *expression in our study is therefore not at odds with the lack of association with BP we observed.

A limitation of the present study is that expression was only tested in white blood cells, rather than in a tissue of potentially greater relevance to BP regulation. However, many *cis*-acting influences on gene expression are expected to be the same in different cell types [28], although tissue-specific differences have been described [29]. In the case of *STK39 *this approach is supported by the fact that rs35929607 genotype correlated with expression in immortalised cell lines derived from cervical tumour cells (HELA) and embryonic kidney cells (HEK293) in the study by Wang and colleagues, as well as with expression in blood in the present study. However, the effects may vary in other tissues. We have analysed the SNPs influencing expression in a South African cohort of mixed ethnicity, but the SNPs associated with expression may vary in different populations due to differing allele frequencies and LD patterns. Although the associations of SNPs with *STK39 *expression that we observe are highly statistically significant, the biological significance of these findings is less certain, since we cannot say what impact such an effect on expression has on disease risk. However, even small differences in gene expression due to genetic factors that are present throughout an individual's lifetime could contribute to differences in common late-onset phenotypes such as hypertension and, as mentioned above, the effects may be greater in tissues related to disease.

Future studies will be necessary to investigate the association of other SNPs at the *STK39 *locus with BP, or to determine whether rare mutations at this locus contribute significantly to population blood pressure variation, as has been shown for other genes implicated in hypertension causation [30].

## Conclusions

*STK39 *expression is modified by polymorphisms acting in *cis*, but there is no evidence that these SNPs affecting *STK39 *transcription are associated with BP in a British Caucasian cohort.

## Competing interests

The authors declare that they have no competing interests.

## Authors' contributions

MSC designed the assays, performed experiments, collected and extracted RNA from the African participants, analysed data and drafted the manuscript. CK performed experiments and assisted with data analysis. PJA and MSK analysed data. BMM supervised sample collection and DNA extraction from the African participants. BK designed the study, ascertained the British families and performed the initial phenotyping and DNA collection. All authors were involved in critical revision of the manuscript. All authors read and approved the final manuscript.

## Pre-publication history

The pre-publication history for this paper can be accessed here:

http://www.biomedcentral.com/1471-2350/10/135/prepub

## Supplementary Material

Additional file 1 **Supplementary methods and tables**. Contains supplementary methods and tables referred to in the text. Click here for file
