# Supplementary material for: STK39 polymorphisms and blood pressure: an association study in British Caucasians and assessment of cis-acting influences on gene expression
Source: BMC Med Genet. 2009 Dec 14;10:135. doi: 10.1186/1471-2350-10-135 (PMC2803166; doi:10.1186/1471-2350-10-135)
Supplement: Additional file 1 — Supplementary methods and tables. Contains supplementary methods and tables referred to in the text. [file 1471-2350-10-135-S1.DOC]

**Additional file 1**

**Supplementary methods**

**Genotyping**

Multiplex SNP genotyping was performed using PCR followed by primer extension and MALDI-TOF mass spectrometry using iPLEX Gold technology from Sequenom (Sequenom Inc, San Diego, USA). SNP assays were designed using Sequenom’s RealSNP (www.RealSNP.com) and MassARRAY Assay Design v3.0 Software.

PCR was performed using 20ng of DNA in a 10µl reaction volume containing 1U HotStar Taq (Qiagen), 100nM each primer (Metabion), 1.25x HotStar PCR Buffer (Qiagen), 500µM dNTP mix (New England Biolabs) and 1.625mM MgCl (Qiagen). Primer sequences are shown in Supplementary Table 2. PCR was performed at 95^o^C for 15 min, followed by 35 cycles of (95^o^C for 20 s, 56^o^C for 30 s, 72^o^C for 1 min), followed by 72^o^C for 1 min. 5µl of the amplification product was added to a 2µl solution containing 0.3U shrimp alkaline phosphatase (Sequenom) and 0.17µl 10x SAP buffer (Sequenom). Samples were incubated at 37^o^C for 20 min followed by 85^o^C for 5 min. 2µl of extension mix containing 0.041µl iPLEX enzyme (Sequenom), 0.2µl iPLEX termination mix (Sequenom), 0.2µl 10x iPLEX buffer (Sequenom), and extension primers (Metabion) with concentrations varying by mass were then added. Extension primers were divided by mass into 4 groups and adjusted to give final concentrations in the 9µl reaction of 0.625µM, 0.83µM, 1.04µM, and 1.25µM for the lowest to highest mass groups respectively. Reactions were incubated at 94^o^C for 30 s, followed by 40 cycles of annealing (94^o^C for 5 s) and extension (5 cycles of 52^o^C for 5 s then 80^o^C for 5 s), followed by 72^o^C for 3 min. Samples were desalted by the addition of 6mg of Clean Resin (Sequenom) in a 25µl reaction and 15nl was dispensed onto a 384 element SpectroCHIP (Sequenom) for MALDI-TOF analysis using SpectroACQUIRE v3.3.1.3 (Sequenom). Spectra were analysed using MassARRAY Typer v3.4 Software (Sequenom).

**Allelic expression**

Quantification of the allelic expression ratio was performed by primer extension and MALDI-TOF mass spectrometry using iPLEX Gold technology from Sequenom (Sequenom Inc, San Diego, USA). PCR was performed using 1µl of cDNA in a 10µl reaction volume containing the same concentrations of reagents as for genotyping described above. PCR conditions were 95^o^C for 15 min, followed by 45 cycles of (95^o^C for 20 s, 58.6^o^C for 30 s, 72^o^C for 1 min), followed by 72^o^C for 1 min. Shrimp alkaline phosphatase and extension reaction steps were performed as described above for genotyping, using the following extension reaction incubation protocol: 94^o^C for 30 s, followed by 30 cycles of annealing (94^o^C for 5 s) and extension (5 cycles of 52^o^C for 5 s then 80^o^C for 5 s), followed by 72^o^C for 3 min. Spectra were analysed using MassARRAY Typer v3.4 Software (Sequenom) and allelic ratios were estimated as the ratios of the area under the peak representing allele 1 to that representing allele 2. Measurements were performed in four replicates. Results from amplification of genomic DNA were used as equimolar reference to normalise the cDNA values. Analyses were performed using the logarithm of the normalised allelic expression ratio.

**Supplementary tables**

**Table S1. Primer sequences**

| **SNP** | **Forward primer** | **Reverse primer** | **Amplicon length** | **Extension primer** |
| --- | --- | --- | --- | --- |
| **rs3754777** | ACGTTGGATGGCCTGAACAAAAATGAGGAC | ACGTTGGATGATCTCGCCTGTTTCACCCAC | 96 | AGGCGTCTCTGGGTCTTTTACT |
| **rs35929607** | ACGTTGGATGCACACTCATGGAATTAAAGG | ACGTTGGATGTCAGAGGGCTCACATTTTGG | 90 | ATGGAATTAAAGGATTATTAGGATACC |
| **rs6749447** | ACGTTGGATGTGGAGTCTGCTAGTACTAGA | ACGTTGGATGCAGTTAGGTCACCTCCTTTC | 100 | GAGTCTGCTAGTACTAGATTAGGA |
| **rs4977950** | ACGTTGGATGCTGAGAACAGTCTTCAACTTG | ACGTTGGATGCACTAGACTGGTTGATTGAA | 100 | CCTGAAGGTTTTTTTTTATATCACTA |
| **rs1061471** | ACGTTGGATGTTTAGAAGTTACAAATACTC | ACGTTGGATGGCTTCTTGCAGTTAATCTCG | 93 | GAAGTTACAAATACTCCAAAGA |
| **rs1802105** | ACGTTGGATGGAAGGCTAATGGCACTTACC | ACGTTGGATGAGAGTACCTGATTGAGAAGC | 100 | CTTTGGGCTATGTCTGGTG |
| **rs56031549** | ACGTTGGATGGATGAGAAGAGCGAAGAAGG | ACGTTGGATGACACAGATTAGCTCATCGTC | 100 | GCGAAGAAGGGAAAGCA |
| **rs56048258** | ACGTTGGATGATTCAAGCCATGAGTCAGTG | ACGTTGGATGGCCAAAGTTCATCTTTGACC | 99 | ATGAGTCAGTGCAGCCA |
| **rs56330212** | ACGTTGGATGGGCACAAGAAGAAGCTTCTC | ACGTTGGATGAACACTGTTTGCTGTTTTC | 96 | TCTGTAGTCTTCATTAGCATT |
| **rs56697518** | ACGTTGGATGACACAGATTAGCTCATCGTC | ACGTTGGATGGATGAGAAGAGCGAAGAAGG | 100 | CGTCAACGATITCATTTAC |

**Table S2. Allele frequency of transcribed SNPs in 309 South African individuals**

| **SNP** | **Alleles*** | **Number genotyped** | **Number of heterozygotes** | **MAF** | **HapMap YRI**  **MAF** | **Hardy-Weinberg**  **p value** |
| --- | --- | --- | --- | --- | --- | --- |
| **rs1061471** | G / A | 298 | 35 | 0.069 | 0.119 | 0.15 |
| **rs1802105** | A / C | 298 | 0 | 0 | NA | NA |
| **rs56031549** | C / T | 298 | 2 | 0.003 | NA | 0.95 |
| **rs56048258** | A / G | 296 | 5 | 0.008 | NA | 0.88 |
| **rs56330212** | G / A | 296 | 0 | 0 | NA | NA |
| **rs56697518** | C / T | 298 | 2 | 0.003 | NA | 0.95 |

***** Major allele given first; MAF, minor allele frequency; YRI, HapMap YRI African cohort; NA, not available.
